# 

**DOI:** 10.1192/bjb.2021.2

**Published:** 2021-06

**Authors:** David Dodwell

**Affiliations:** locum consultant psychiatrist with Cambridgeshire and Peterborough NHS Foundation Trust, UK. Email: david.dodwell@cpft.nhs.uk

Ruth Paddon has written a short biography. It is devoid of self-pity and rancour; it has a simple, pellucid style that I found very moving.

She describes coming through childhood abuse, anxiety and parental illness to reach an early adulthood that appeared promising. She then developed depression and psychosis (and anorexia) and had several hospital admissions. Her initial life plans and expectations were not fulfilled but she made use of other options that became available, including moving to Italy. Adverse experiences are clear but not harrowing, there is no bitterness despite the pain, and she thanks those who have supported her. The personal descriptions are complemented by family photos which locate her and her family as ordinary human beings.

As a psychiatrist, I often reflect on how little time I spend with each patient: maybe 15 minutes a week for an in-patient and 30 minutes every 3 months for an out-patient. Meanwhile, they are hearing voices or experiencing negative thoughts most of the time every day. Paddon gives a very balanced view: it is clear that most of her time is without professionals but she is positive about all the staff she mentions; she values the specific role they have to play (particularly when performed with warmth and friendliness). She is clear about the more extensive (and different) help she has had from her family (particularly siblings).

I knew Ruth Paddon in a professional capacity many years ago. I found it personally educational to see how I figured (briefly) in her life and compare it with my experience of being her psychiatrist.

This book is very readable and it is relatively short (34 pages of text and 10 of photos). I would recommend it to psychiatric staff, patients and relatives equally. It is a balanced, genuine and accessible account of a life that has been intermittently diverted or temporarily held up (the ‘stillness’ of the title) by depression and psychosis.

It is currently available only to order from the publisher (email: info@tempoallibro.it).

